# Editorial: Applications of medicinal plants and their metabolites in fibrotic disease: novel strategies, mechanisms, and their impact on clinical practice

**DOI:** 10.3389/fphar.2025.1711009

**Published:** 2025-11-07

**Authors:** Houfu Qian, Xiang Tao, Li Yuan, Xin Wang, Bing Yu

**Affiliations:** 1 Department of Gastroenterology, Taizhou Second People’s Hospital, Taizhou, Jiangsu, China; 2 Department of Cell Biology, Naval Medical University, Shanghai, China; 3 Department of Pathology, Obstetrics and Gynecology Hospital of Fudan University, Shanghai, China; 4 Department of Nephrology, Affiliated Hospital of Nantong University, Nantong, China; 5 Molecular and Cellular Therapeutics, University of Minnesota Twin Cities, StPaul, United States

**Keywords:** fibrosis, natural products, traditional Chinese medicine, medicinal plants, drug development

Fibrotic diseases represent a major global health challenge. They are pathologically characterized by the excessive deposition of the extracellular matrix, which ultimately leads to organ dysfunction and failure (Zhao et al., 2022; Kim T. R. et al., 2025). Current therapeutic options remain severely limited, with only a few anti-fibrotic agents approved for clinical use. Examples include pirfenidone and nintedanib for idiopathic pulmonary fibrosis (IPF) and the more recently approved resmetirom for non-alcoholic steatohepatitis (NASH)-related liver fibrosis (Ahangari et al., 2022; Harrison et al., 2024). However, the utility of these drugs is considerably constrained by issues including significant gastrointestinal side effects and poor patient tolerance, which markedly limit their clinical efficacy and long-term adherence. In response to these challenges, natural products derived from medicinal plants and fungi, with their innate “structural diversity–multi-components–multi-targets” synergy, excellent biocompatibility, and safety profiles, have emerged as a highly promising new direction in the research on therapies for fibrotic diseases (Kuete et al., 2025; Li et al., 2025).

This Research Topic presents eight insightful contributions (Table 1) that collectively advance our understanding of the molecular mechanisms and therapeutic potential of natural compounds in diverse fibrotic contexts, spanning liver, lung, kidney, intestinal, and systemic fibrotic diseases. Each study highlights novel strategies and pathways, reinforcing the translational promise of medicinal plants and their metabolites in fibrosis management.

**TABLE 1:** Overview of the eight featured articles in this Research Topic.

Article Type	Fibrosis model	Species	Natural products	Key findings	References
Research Article	Carbon tetrachloride-induced hepatic fibrosis	Rat	rhaponticin (RHA)	• RHA alleviates hepatic fibrosis by improving liver function, inhibiting the activation of hepatic stellate cells, and reducing collagen deposition • RHA modulates glycerophospholipid, phenylalanine, and tryptophan metabolism, suggesting a multi-target mechanism	Yang et al.
Review	Idiopathic Pulmonary Fibrosis (IPF)		More than 20 natural products, such as flavonoids, saponins, polyphenols, terpenoids, natural polysaccharides, cyclic peptides, deep-sea fungal alkaloids, and algal proteins	• Summarizes the pathological mechanisms of IPF, including epithelial injury, fibroblast transformation, extracellular matrix (ECM) dysregulation, and the roles of the TGF-β/Smad and NF-κB signaling pathways • Delineates the multi-pathway anti-fibrotic effects of over 20 natural products • Discusses the challenges hindering the clinical application of these natural products, alongside emerging technologies that offer potential solutions	Ma et al.
Research Article	Adenine-induced renal fibrosis (chronic kidney disease)	Mouse	Jingtian Granule (JT)	• JT alleviates renal fibrosis by activating the mitochondrial deacetylase SIRT3, which mediates the deacetylation of P53, thereby inhibiting ferroptosis and lipid peroxidation	Xiong et al.
Review	Inflammatory bowel disease (IBD)-related intestinal fibrosis			• Elucidates the mechanisms of intestinal fibrosis, involving inflammatory cells, pro-fibrotic cytokines, and gut microbial metabolites • Explores integrated management strategies, including traditional Chinese medicine, stem cell therapy, and lifestyle interventions such as dietary changes and vitamin D supplementation	Li et al.
Research Article	Bleomycin-induced pulmonary fibrosis (a model for Connective Tissue Disease-Associated Interstitial Lung Disease, CTD-ILD)	Mouse	polydatin (PD) + curcumin (Cur)	• The combination therapy of PD and Cur (PD + Cur) ameliorates pulmonary fibrosis by activating the GABBR receptor, modulating the PI3K/AKT/TGF-β pathway, and consequently inhibiting immune cell activation and fibrogenesis	Zhang et al.
Review	Liver fibrosis		Biomedicine medicines, Repurposed drugs, Protein and nucleic acid molecules, Botanical drug metabolites, and Traditional Chinese medicine	• Summarizes the mechanisms of liver fibrosis and antifibrotic pharmacotherapies • Highlights the potential of natural products, combination therapies, and nanotechnology • Points out the challenges in clinical translation (such as gaps in standardized assessment and large-scale validation, which necessitate clinical trials)	Chen et al.
Review	Fibrosis in Multiple Organs (Lung, Heart, Kidney, Liver, Bladder, Skin, and Arachnoid)		Icariin (ICA) and related metabolites	• Summarizes the anti-fibrotic mechanisms of ICA in multiple organ fibrosis (including anti-inflammatory, antioxidant, and mitochondrial regulation, along with modulation of multiple pathways such as TGF-β/Smad and NF-κB) • Discusses the challenges and potential future directions (bioavailability challenges and nano-drug delivery systems; the need for clinical trial validation)	Zhao et al.
Research Article	Diethylnitrosamine (DEN)-induced hepatic fibrosis	Mouse	Fangchinoline (FAN)	• FAN alleviates hepatic fibrosis by inhibiting stellate cell activation, modulating taurine metabolism (restoring CSAD enzyme and taurine levels), reducing ROS, and activating the Nrf2 pathway • Taurine and NAC synergistically enhance the effect of anti-hepatic fibrosis	Yin et al.

The study by Yang et al. demonstrates that rhaponticin (RHA) alleviates liver fibrosis in a carbon tetrachloride-induced rat model by improving liver function, inhibiting the activation of hepatic stellate cells, and reducing collagen deposition. Non-targeted metabolomics revealed the modulation of multiple metabolic pathways, particularly those of glycerophospholipids, phenylalanine, and tryptophan, suggesting a multi-targeted mechanism underlying RHA’s therapeutic effects. These findings not only provide robust evidence supporting RHA as a promising natural product for anti-liver fibrosis therapy but also deepen our understanding of the metabolic mechanisms underlying liver fibrosis, offering important insights for future research in this field.

The review by Ma et al. offers a comprehensive and insightful overview of idiopathic pulmonary fibrosis (IPF). It integrates key pathogenic mechanisms, such as epithelial injury, fibroblast-to-myofibroblast transition, ECM dysregulation, and key signaling cascades including TGF-β/Smad and NF-κB, with a systematic survey of over 20 anti-fibrotic natural products from terrestrial and marine sources. A particular strength of this article lies in its organization of diverse natural compounds, including flavonoids, saponins, polyphenols, and marine alkaloids, according to their multi-target mechanisms, which helps clarify their therapeutic relevance. Notably, the authors do not shy away from addressing translational challenges, such as bioavailability and mechanistic complexity, while also outlining how advanced tools such as high-throughput screening and Artificial Intelligence (AI)-assisted drug design could accelerate future discovery. By bridging molecular pathogenesis, natural product pharmacology, and cutting-edge technology, this review offers a structured and forward-looking perspective that will be valuable for researchers exploring novel IPF therapeutics.

The study by Xiong et al. offers valuable insights into the anti-fibrotic mechanism of Jingtian Granule (JT) in chronic kidney disease. Using an adenine-induced mouse model, the authors demonstrate that JT ameliorates renal fibrosis through the activation of the mitochondrial deacetylase SIRT3, which deacetylates the tumor suppressor P53, thereby suppressing ferroptosis and lipid peroxidation. SIRT3 knockout abrogated JT’s protective effects, confirming its pivotal role. *In vitro* experiments with HK-2 cells corroborated these findings by modulating ROS and the SIRT3/P53/GPX4 axis. Importantly, this research bridges traditional Chinese medicine with modern cell death mechanisms, revealing a novel link between ferroptosis inhibition and renal anti-fibrotic therapy. The identification of the SIRT3/P53 axis not only elucidates a sophisticated molecular pathway but also highlights its potential as a therapeutic target for CKD treatment.

The review by Li et al. provides a comprehensive analysis of the cellular and molecular mechanisms driving intestinal fibrosis in inflammatory bowel disease (IBD). It effectively elucidates the roles of inflammatory cells (eosinophils, mast cells, macrophages), profibrotic cytokines (IL-17, IL-6, IL-34), and gut microbiota metabolites in fibrosis progression. The authors also examine current and potential therapeutic agents, including mesenchymal stem cells, PPAR-γ agonists, and pirfenidone, with an emphasis on traditional Chinese medicine and its multi-target advantages. Furthermore, lifestyle interventions such as dietary modulation and vitamin D supplementation are proposed as adjunctive strategies, advocating integrative approaches to IBD-related fibrosis management. By bridging molecular mechanisms with holistic treatment approaches, this review makes a valuable contribution to the evolving paradigm of IBD-related fibrosis therapy.

The study by Zhang et al. presents a compelling investigation into the anti-fibrotic synergistic effects of polydatin and curcumin (PD + Cur) in a bleomycin-induced model of connective tissue disease-associated interstitial lung disease (CTD-ILD). Using multi-omics approaches, the authors convincingly demonstrate that PD + Cur alleviates alveolar damage and fibrosis by activating GABBR, leading to downstream suppression of the PI3K/AKT/TGF-β signaling axis. Further pharmacological validation using GABBR agonists and antagonists solidifies the mechanistic link between target engagement and therapeutic outcome. These findings not only reveal a novel role for GABBR in fibrotic lung disease but also highlight the promise of natural product combinations as multi-target strategies for treating CTD-ILD.

The review by Chen et al. provides an authoritative overview of pharmacological strategies for liver fibrosis, systematically cataloging interventions from conventional drugs, repurposed drugs, plant-derived metabolites, and traditional Chinese formulas. Notably, the authors highlight promising advanced approaches, such as combination therapies, extracellular vesicles, and nanotechnology, for enhancing targeting precision. While acknowledging encouraging preclinical and clinical results, the review also critically identifies persistent gaps, including the lack of standardized efficacy assessment and large-scale validation. By emphasizing the need for rigorous clinical trials and improved translational frameworks, this work constructively outlines a pathway to bridge the gap between natural product research and clinically applicable liver fibrosis therapies.

The review by Zhao et al. comprehensively analyzes icariin (ICA) as a multi-organ anti-fibrotic agent, covering fibrosis in the lung, heart, kidney, liver, bladder, skin, and arachnoid membrane. The authors delineate ICA’s pleiotropic mechanisms, which range from anti-inflammatory and antioxidant effects to the regulation of mitochondrial function, cell death, autophagy, and macrophage polarization, through key pathways, including TGF-β/Smad, NF-κB, AMPK, Nrf2/HO-1, and WNT/β-catenin. The authors not only present compelling preclinical evidence but also critically address pharmacokinetic limitations, highlighting advanced extraction methods and nano-delivery strategies to overcome bioavailability challenges. While the preclinical profile is notably robust, the review appropriately emphasizes the crucial need for clinical validation. This contribution successfully positions ICA as a promising multi-target natural product while constructively outlining the translational pathway from mechanistic understanding to potential clinical application.

The study by Yin et al. presents a mechanistically insightful investigation into the anti-fibrotic properties of fangchinoline (FAN) in a diethylnitrosamine-induced liver fibrosis model. The authors demonstrate that FAN attenuates liver injury and collagen deposition by inhibiting stellate cell activation and extracellular matrix protein expression, and also reveal that FAN regulates taurine metabolism by specifically restoring CSAD enzyme expression and hepatic taurine levels. Taurine synergistically enhances FAN’s anti-fibrotic effect by reducing reactive oxygen species and activating the Nrf2 antioxidant pathway. Combined treatment with the ROS scavenger N-acetylcysteine further potentiated these effects. Thus, this study not only delineates a taurine-mediated mechanism for FAN but also highlights the therapeutic potential of targeting metabolic pathways in liver fibrosis.

As a multifaceted pathological process driven by numerous factors and signaling cascades, fibrosis inherently necessitates multi-target therapeutic strategies (Antar et al., 2023). The findings presented in this Research Topic strongly support the role of medicinal plants and their bioactive metabolites as promising sources for such interventions. These natural agents demonstrate the ability to concurrently modulate key processes in fibrosis, including metabolic reprogramming, ferroptosis, immune responses, oxidative stress, and critical signaling pathways, thereby validating their potential for comprehensive therapeutic intervention. This multi-target capacity represents a distinct advantage over single-target agents, because it aligns more closely with the complex pathogenesis of fibrotic diseases.

However, the current body of evidence remains largely confined to preclinical studies, characterized by limited sample sizes and a predominant reliance on *in vitro* and animal models. The critical absence of clinical trial data constitutes a major bottleneck to translation. Further complicating this transition are the inherent challenges of natural products, such as suboptimal physicochemical properties (e.g., poor solubility, low bioavailability, and a short half-life), complex pharmacokinetics, and difficulties in standardization (Kim H. K. et al., 2025; Liu H. et al., 2025; Liu X. et al., 2025).

To bridge this gap, a convergent research paradigm is urgently needed. Future work should integrate traditional medical knowledge with advanced technological platforms—such as multi-omics, nanomedicine, and AI—to optimize drug delivery, validate mechanisms of action, and ultimately power rigorous clinical trials. We hope this Research Topic will inspire innovative approaches that bridge ethnopharmacology and modern science, ultimately advancing effective anti-fibrotic therapies into clinical practice.
